# Doxepin as OCT2 inhibitor ameliorates inflammatory response and modulates PI3K/Akt signaling associated with cisplatin-induced nephrotoxicity in rats

**DOI:** 10.1007/s00210-024-03473-1

**Published:** 2024-10-14

**Authors:** Mariam H. Fawzy, Yasser M. Moustafa, Dina M. Khodeer, Noha M. Saeed, Norhan M. El-Sayed

**Affiliations:** 1 Pharmacology and Toxicology Department, Faculty of Pharmacy, Egyptian Russian University, Cairo, Egypt; 2 Pharmacology and Toxicology Department, Faculty of Pharmacy, Suez Canal University, Ismailia, 41522 Egypt; 3 Department of Pharmacology & Toxicology, Faculty of Pharmacy, Badr University in Cairo, Badr City, Egypt

**Keywords:** Apoptosis, Cisplatin, Doxepin, OCT2, PI3K/Akt

## Abstract

Organic cationic transporter 2 (OCT2) was identified as the main transporter involved in the accumulation of cisplatin (CP) in the proximal tubular renal cells, resulting in nephrotoxicity. Doxepin (DOX) is a tricyclic agent with an inhibitory effect on OCT2. This study aimed to explore the possible mechanisms of the renoprotective role of DOX toward CP-induced nephrotoxicity. Rats were randomly divided into six groups: group 1, control; group 2, CP; groups 3, 4, and 5 were treated with graded doses of DOX (5, 10, and 20 mg/kg, respectively) intraperitoneally (ip) once daily for 10 consecutive days and group 6 was treated only with DOX (20 mg/kg). On the seventh day, a single injected dose of CP (10 mg/kg, ip) was given to the rats in groups 2–5. Seventy-two hours after CP injection, rats were sacrificed, and the kidneys were removed for histological and biochemical measurements. DOX ameliorated the CP-induced histopathological alterations. DOX significantly reduced the expression of OCT2, lipid peroxidation marker (MDA), and inflammatory cytokines, including TNF-α, IL-6, IL-1, IL-2, and IL-1β, and increased the activity of antioxidant enzymes. In addition, pre- and co-treatment with DOX significantly reduced the CP-mediated apoptotic effect by reducing the renal tissue expression of BAX and caspase-3 levels, upregulating the expression of Bcl-2, and modulating the phosphorylation of PI3K/Akt signaling cascade. DOX exerts a nephroprotective impact against CP-mediated nephrotoxicity via the inhibition of OCT2, suppression of inflammation, oxidative stress, and apoptotic markers, and modulation of PI3K/Akt signaling cascade.

## Introduction

Nephrotoxicity greatly hinders the exploitation of cisplatin (CP) therapy, which is a powerful and irreplaceable agent in the cancer field. CP exerts a substantial and influential efficiency in the management of diverse malignant disorders (Karasawa and Steyger 2015). The attempts currently being adopted to mitigate or prevent the toxicity caused by CP include hydration, forced diuresis using mannitol, or magnesium (Crona et al. 2017). However, these interventions are not enough to prevent CP-induced nephrotoxicity. Therefore, researchers look for other safe alternative nephroprotective drugs without compromising the antineoplastic effect of CP (Volarevic et al. 2019).

The uptake of CP in the kidneys is mediated by passive diffusion or facilitated diffusion (Gómez-Sierra et al. 2018; Pabla and Dong 2008). Organic cation transporter 2 (OCT2) is known to be a member of the organic cation transporter family that involves three isoforms: OCT1, OCT2, and OCT3 (Yonezawa et al. 2005). It was reported that there is a strong association between OCT2 and the selective toxicity of CP, which is exhibited especially in the S3 segment of proximal tubular epithelial cells, which is characterized by high OCT2 expression (Ciarimboli 2014; Ozkok and Edelstein 2014). Further, it was found that OCT2-deficient mice were protected from CP-induced nephrotoxicity (Peres and Cunha Júnior 2013). This could also be illustrated clearly in diabetic rats that were resistant against CP-induced nephrotoxicity and showed a decreased OCT2 expression (Grover et al. 2002). Furthermore, in another study, the nephroprotective action of imatinib has been attributed mainly to its ability to inhibit OCT2-mediated renal accumulation of CP (Tanihara et al. 2009). Thus, OCT2 inhibition may contribute in ameliorating CP-induced nephrotoxicity.

Additionally, CP is postulated to directly initiate oxidative stress and contribute to the depletion of the antioxidant protection system either via its interaction with glutathione (GSH) or through its effect on the respiratory chain of mitochondria, resulting in the production of excessive reactive oxygen species (ROS), aggravating cellular stress (McSweeney et al. 2021). ROS has been recognized as the initial event for the production of tumor necrosis factor-alfa (TNF-α), which is primarily produced by renal cells. TNF-α is linked to the activation of other proinflammatory cytokines, such as interleukin (IL-1, IL-6, IL-1β), and (IL-2), which have also been associated with CP-induced nephrotoxicity (Volarevic et al. 2019; Yan et al. 2017). Moreover, the release of glycoprotein myeloperoxidase (MPO) in response to the activation of neutrophils has also been implicated with CP-triggered nephrotoxic effects (Karadeniz et al. 2011).

ROS also plays another role in the initiation of apoptotic renal tubular cell damage, which has been illustrated by the activation of Bcl-2 family proteins and has been linked to CP-mediated nephrotoxic effects (Li et al. 2020). CP administration is followed by the activation of pro-apoptotic protein (BAX), which plays a role in the release of additional apoptogenic factors; which in turn, ends with caspase-3 activation (Peres and Cunha Júnior 2013; McSweeney et al. 2021). This is associated with the decrease in the antioxidant protein B-cell lymphoma 2 (Bcl-2) levels (Rjiba-Touati et al. 2012). This alteration in the expression of apoptogenic proteins accounts for the triggering of mitochondrial and apoptotic renal tissue damage (Jiang and Dong 2008; Jiang et al. 2006).

In addition, triggering the phosphatidylinositol 3-kinase (PI3K)/Akt cascade has also been associated with CP-induced nephrotoxicity (Miao and Degterev 2011). The PI3K/Akt pathway plays a chief role in keeping the survival of renal cells via the induction of antioxidant response element expression (Zhou et al. 2019), suppressing the surge of inflammatory mediators, and modulating the apoptotic machinery by upstreaming the pro-survival Bcl-2 protein (Thangapandiyan et al. 2019).

Doxepin hydrochloride (DOX), which is a tricyclic antidepressant agent, can inhibit the reuptake of serotonin and norepinephrine, is histaminergic, and has alpha-1adrenergic antagonistic effects (Edmonds and Swanoski 2017). DOX can also inhibit OCT2 transporters (Hacker et al. 2015). This study aimed to look into the possible renoprotective function of DOX as an OCT2 inhibitor versus CP-induced nephrotoxicity and determine its critical antioxidant, anti-inflammatory, and anti-apoptotic effects via the modulation of the PI3K/Akt pathway.

## Materials and methods

### Drugs and chemicals

CP and DOX were obtained from (Sigma, St. Louis, MO). CP and DOX were dissolved in 0.9% saline.

### Animals

Male Wister albino rats (8 weeks old) were acquired from Nile Co. for pharmaceutical and chemical industries (Egypt). The animals were kept in well-ventilated cages at room temperature (25 °C) under controlled light cycles (12 h light/12 h dark). They were fed with a standard pellet chow diet and allowed free access to tap water. The animals were kept for at least 2 weeks prior to the experiment for acclimatization. All animal procedures were done in accordance with the protocol approved by the ethics committee of Faculty of Pharmacy, Suez Canal University, 201911PHDA2.

### Experimental design

Thirty-six rats were randomly divided into six groups of six animals in each:

- The first group: rats were administrated 0.9% saline intraperitoneally (ip) daily for 10 consecutive days and were considered as the control (**CON**) group.
- The second group: rats were administrated (0.9% saline, ip) daily for 10 consecutive days and were received a single injection of CP (10 mg/kg, ip) on the seventh day of the experiment. This was considered as the CP-treated group (CP) (El-Sayed et al. 2021).
- The third group: rats were received DOX (5 mg/kg, ip) daily for 10 days (Azadbakht et al. 2015) and were received a single injection of CP (10 mg/kg, ip) on the seventh day of the experiment. This group was denoted as (CP + DOX5).
- The fourth group: rats were received DOX (10 mg/kg, ip) daily for 10 days (Azadbakht et al. 2015) and were received a single injection of CP (10 mg/kg, ip) on the seventh day of the experiment. This group was denoted as (CP + DOX10).
- The fifth group: rats were received DOX (20 mg/kg, ip) daily for 10 days (Wrzosek et al. 2009) and were received a single injection of CP (10 mg/kg, ip) on the seventh day of the experiment. This group was denoted as CP + DOX20.
- The sixth group: rats were only received DOX (20 mg/kg, ip) for 10 days and were considered the DOX-treated group DOX20 only.

Following the CP administration on the eighth day, all groups of rats were kept in metabolic cages for 72 h soon before anesthesia to measure the urine production. Then, the animals were sacrificed under anesthesia using 100 mg/kg of ketamine and 10 mg/kg of xylazine ip. Blood was drawn from the retro-orbital plexus and was centrifuged to separate the serum, which was separated into aliquots and stored at (− 80 °C) until further analysis. The kidney was removed through dissection. For further investigation, a portion of the kidney was immersed in liquid nitrogen and preserved at (− 80 °C), while the remaining portion was removed and preserved in 10% phosphate-buffered formalin for histological analysis.

### Histopathological examination

For 48 h, 10% neutral buffered formalin was used to fix the kidney tissue samples. Afterwards, the samples were infiltrated and implanted in a paraplast tissue embedding medium after being treated in consecutive grades of ethanol and cleared in xylene. A rotatory microtome was used to cut these samples into 4 µm-thick tissue sections, which were subsequently mounted on glass slides. Then, for general morphological examination, the tissue sections were stained with Hematoxylin and Eosin and were observed under a light microscope (Leica Microsystems GmbH, Wetzlar, Germany), and the magnification power used was (40 ×). All conventional processes of sample fixing and staining were in accordance with Culling (Culling 1974).

### Spectrophotometric method

Spectrophotometric procedures were used to evaluate the kidney function indicators; serum creatinine (Scr), blood urea nitrogen (BUN), and albumin levels were assessed using commercially available kits [Cat. No. CR1250, Biodiagnostic Co., EL Dokki St, Egypt], [Cat. EIABUN, Thermo Fisher Scientific, Waltham, MA, USA], and [Cat. No. AB1010, Biodiagnostic Co., EL Dokki St, Egypt], respectively. The results are expressed as mg/dL.

### Colorimetric assessment

MDA (Cat. No. MDA2528), GSH (Cat. No. GR 2511), and SOD (Cat. No. SD2520) levels were assessed in renal tissue homogenates as indicators of lipid peroxidation status and antioxidant defense activity, respectively, using the purchased commercially available kits, following the manufacturer’s instructions. The results for MDA, GSH, and SOD are expressed as nmol/mg protein, mmol/mg, and U/mg, respectively.

### Enzyme linked immunosorbent assay

The Sandwich ELISA technique was used to detect the tissue levels of TNF-α (Cat. No. CSB-E11987, Cusabio, Fannin St., Houston, USA), IL-6 (Cat. No. R6000B, R&D Systems, Canada), IL-2 (Cat. No. CSB-E04628r, Cusabio, Fannin St., Houston, USA), IL-1β (Cat. No. MBS825017, Mybiosource, Inc. Co., San Diego, USA), and MPO (Cat. No. RDR-MPO-Ra, Biotech, Inc., Alpharetta, GA, USA) as indicators of the inflammatory state, following the manufacturers’ instructions. The results are expressed as pg/mg protein.

### Western blot analysis

A RIPA lysis buffer was used to lyse the renal tissue samples and extract all proteins. Cell debris was removed by centrifugation at 16,000 × g for 30 min at 4 °C after storing the lysate on ice for 30 min on a shaker. Then, the proteins in the supernatant were collected and transferred to a separate tube for further measurement of protein concentration. For quantitative protein analysis, the Bradford Protein Assay Kit (SK3041) (Bio Basic Inc. Markham, Ontario L3R 8T4, Canada) was utilized. Then, 20 µg of protein in each sample was added with an equivalent volume of Laemmli sample buffer. Each previous mixture was heated to 95 °C for 5 min to denaturize the protein before putting them onto polyacrylamide gels for electrophoresis. The membranes were blocked in tris-buffered saline with Tween 20 (TBST) buffer and 3% bovine serum albumin for 1 h at room temperature. Then, the membranes were incubated overnight at 4 °C with the following primary antibodies: OCT2 (0.5 µg/mL, Cat. No. PA5-80015, Thermo Fisher Scientific, USA), anti-Akt (Cat. No. PA5-77855, Thermo Fisher Scientific, 1:1000 dilution), anti-phospho-(p)-Akt (1:1000, Cat. No. 600–401-268, Thermo Fisher Scientific, USA), anti-PI3K (1:1000, Cat. No. PA5-99518, Thermo Fisher Scientific, USA), anti-phospho-(p)-PI3K (1:1000, Cat. No. PA5-38905, Thermo Fisher Scientific), and anti-B-actin (1:1000, Cat. #PA1-183, Thermo Fisher Scientific). Then, after washing in TBS, the membranes were probed with HRP-conjugated secondary antibodies (goat anti-rabbit IgG HRP l mg Goat mab, Novus Biologicals, Colorado, USA) for 1 h at room temperature. A chemiluminescent substrate (ClarityTM Western ECL substrate, BIO-RAD, USA Cat. No. 170–5060) was used to quantify and develop protein bands, and chemiluminescent signals were collected using a CCD camera-based imager. After normalization using β-actin, the band intensities of the expressed target proteins were identified using a Chemi DocTM MP imager system.

### Immunohistochemistry

To detect the BAX, Bcl-2, and caspase-3 expression in renal tissues, immunohistochemical staining was done, following the manufacturer’s protocol. A prepared paraffin was used to embed 5 µm-thick renal tissue sections. Deparaffinized recovered tissue slices were treated with 0.3% H_2_O_2_ for 20 min. Then, these were incubated overnight at 4 °C with BAX (Cat. No. MA5-14003, Thermo Fisher Scientific), Bcl-2 (Cat. No. PA1-30411, Thermo Fisher Scientific), and anti-Caspase 3 (Cat. No. RB-1197-R7, Thermo Fisher Scientific) primary antibodies diluted at 1:100, according to manufacturer directions and instructions. The tissue slices were rinsed with PBS and treated with HRP-linked secondary antibodies (Envision kit, DAKO) for 20 min; then, these were washed and incubated with diaminobenzidine for 15 min, as directed by the manufacturer. The samples were washed with PBS, counter-stained with hematoxylin, dehydrated, and cleaned in xylene in cover slides for microscopic analysis. Lastly, to determine the area percentage of BAX, Bcl-2, and caspase-3 immunohistochemistry expression levels in the stained sections, six non-overlapping fields were randomly chosen and scanned from striatal regions of each sample. Eventually, all light microscopic examinations and data were collected using a Leica Application module for histological analysis, which was connected to a Full HD microscopic imaging system (Leica Microsystems GmbH, Germany).

### Statistical analyses

All data are expressed as mean ± SD. The differences among the groups were analyzed by one-way analysis of variance (ANOVA), followed by the Tukey-Kramer test as a post-hoc test. The differences were considered significant at *p* < 0.05. All analyses and graphs were performed and sketched using a Graph Pad prism (ISI® Software, USA) version 5 software.

## Results

### Doxepin ameliorates kidney dysfunction

In this study, 72 h after injecting a single dose of CP, there was a significant increase in BUN, Scr, and urinary volume levels by sevenfold, 6.6-fold, and 3.2-fold, respectively, whereas there was a significant decline in albumin level by 62% as compared to the CON group. In contrast, the co-treatment with DOX at doses 5, 10, and 20 mg/kg exhibited a significant decrease in BUN levels by 17%, 29%, and 55%, respectively, in Scr levels by 27%, 53%, and 72%, respectively, and in urinary volume by 19%, 31%, and 60%, respectively. There was also a significant increase in albumin levels by 1.3-fold, 1.7-fold, and 2.4-fold, respectively, as compared to the CP group in a dose-dependent manner. However, there was no significant change in levels of BUN, Scr, albumin, and urinary volume between CON and Dox 20 only treated group. Moreover, there was a marked reduction in BUN and urinary volume levels by 46% and 50%, respectively, in rats co-treated with 20 mg/kg DOX as compared to those in rats co-treated with 5 mg/kg DOX. Meanwhile, the co-treatment with 20 mg/kg DOX significantly declined the BUN and urinary volume levels by 37% and 1.4-fold, respectively, as compared to those in rats co-treated with 10 mg/kg DOX. Further, there was a marked drop in Scr levels by 36% and 63% in rats co-treated with 10 and 20 mg/kg DOX, respectively, as compared to those in rats co-treated with 5 mg/kg DOX. The co-treatment with (20 mg/kg) DOX significantly declined the Scr levels by 42% as compared to those in rats co-treated with 10 mg/kg DOX. On the other hand, there was a marked elevation in albumin levels by 1.9-fold in rats co-treated with DOX (20 mg/kg) as compared to that in rats treated with DOX (5 mg/kg). The co-treatment with 20 mg/kg DOX significantly raised the albumin levels by 1.4-fold as compared to the co-treatment with 10 mg/kg DOX (Table 1).

**Table 1:** Ameliorating effects of DOX on renal function, oxidative stress, and antioxidant profile indicators

Treated groups	BUN (mg/dl)	Scr (mg/dl)	Albumin (mg/dl)	Urinary volume (ml/24 h)	MDA (nmol/mg)	GSH (mmol/mg)	SOD (U/mg)
CON	21.20 ± 5.20	0.427 ± 0.08	4.178 ± 0.35	8.893 ± 2.423	20.70 ± 3.78	128.2 ± 4.478	65.40 ± 3.67
CP	154.6 ± 20.86^a^	2.818 ± 0.45^a^	1.573 ± 0.37^a^	28.02 ± 1.33^a^	98.23 ± 5.78^a^	50.05 ± 4.22^a^	20.75 ± 2.76^a^
CP + DOX5	128.8 ± 6.60^a,b^	2.067 ± 0.31^a,b^	1.998 ± 0.46^a^	22.72 ± 2.26^a,b^	58.92 ± 8.33^a,b^	100.4 ± 4.46^a,b^	37.05 ± 4.71^a,b^
CP + DOX10	109.8 ± 16.73^a,b^	1.333 ± 0.33^a,b,c^	2.687 ± 0.53^a,b^	19.24 ± 2.62^a,b^	49.75 ± 3.85^a,b,c^	111.6 ± 4.76^a,b,c^	43.30 ± 2.58^a,b,c^
CP + DOX20	69.10 ± 16.60^a,b,c,d^	0.7800 ± 0.16^b,c,d^	3.743 ± 0.55^b,c,d^	11.35 ± 2.48^b,c,d^	33.45 ± 3.82^a,b,c,d^	121.0 ± 5.19^b,c,d^	53.85 ± 2.74^a,b,c,d^
DOX 20 only	21.52 ± 2.91^b,c,d^	0.4867 ± 0.12^b,c,d^	3.925 ± 0.20^b,c,d^	8.287 ± 1.12^b,c,d^	25.05 ± 3.63^b,c,d^	130.5 ± 4.50^b,c,d^	69.75 ± 3.50^b,c,d^

Rats were received DOX (5, 10, 20 mg/kg, ip) for 10 days. On the 7th day, rats were administrated a single injection dose of CP (10 mg/kg, ip). Data are represented as mean ± SD (*n* = 6). a, b, c, or d: significant difference from CON, CP, DOX5, or DOX 10 groups, respectively at *p* < 0.05 using ANOVA followed by Tukey–Kramer as a post-hoc test. *CON* control, *CP* cisplatin, *DOX* doxepin, *BUN* blood urea nitrogen, *Scr* serum creatinine, *MDA* malondialdehyde, *GSH* glutathione, *SOD* superoxide dismutase

### Histopathological examination of renal tissues

**Fig. 1:**
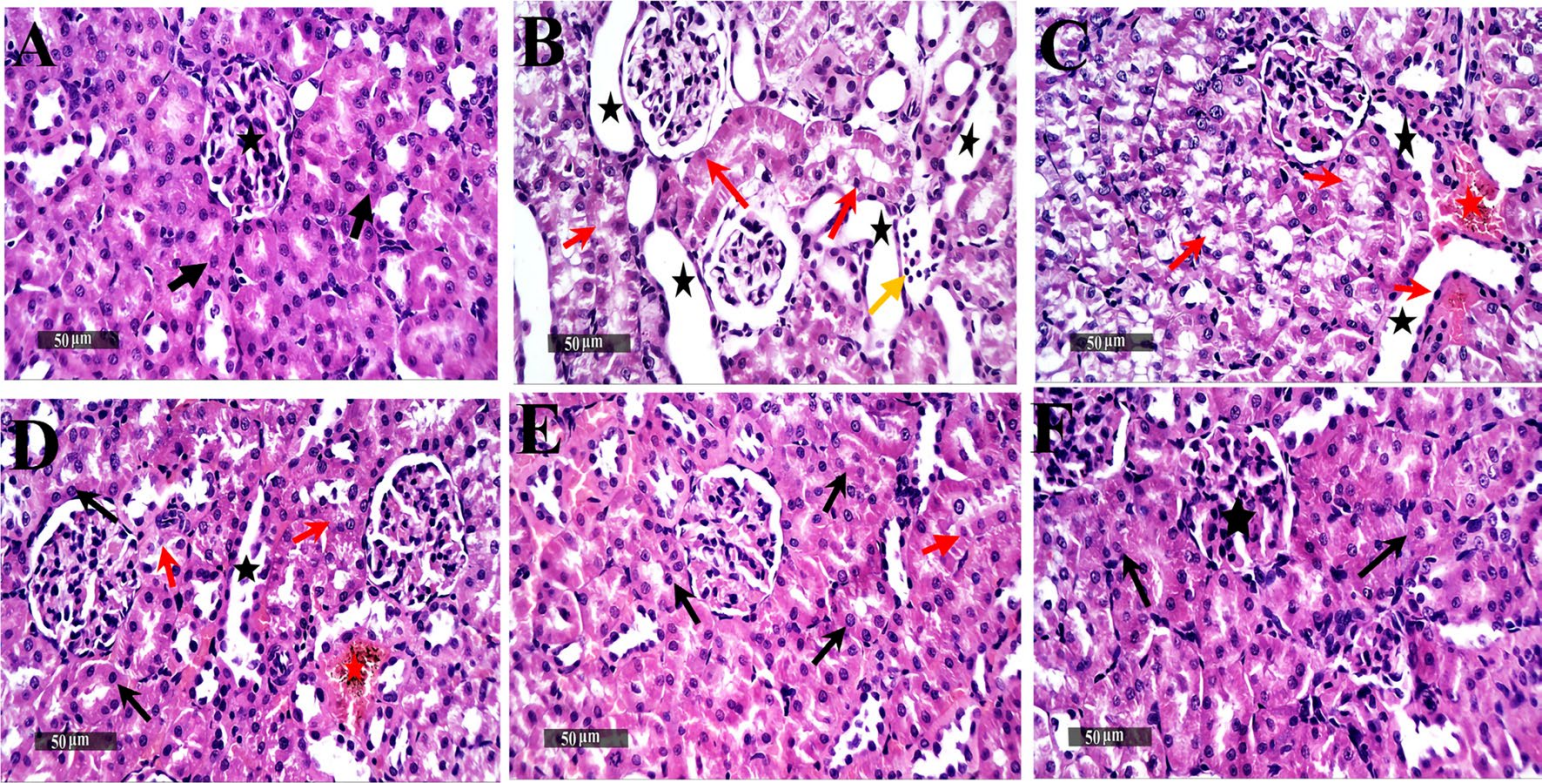
Microscopic examination of renal tissue specimens stained with hematoxylin and eosin. **A** Section taken from a rat in the CON group showing normal histological features of renal parenchyma (black star), different nephron segments with intact tubular epithelium lining (arrow), and intact vasculatures. **B** Section taken from a rat in the CP group showing diffuse necrotic and degenerative changes, including nephron tubular segments (red arrow) with many figures of nuclear pyknosis, occasional intraluminal eosinophilic casts (dashed arrow), moderate tubular dilatation (star), and focal interstitial inflammatory cell infiltrates (yellow arrow). **C** Section taken from a rat in the CP + DOX5 group showing minimal protective efficacy, with persistent records of degenerative changes in the tubular epithelium (red arrow), mild tubular dilatation (black star), and many congested and dilated intratubular BVs (red star). **D** Section taken from a rat in the CP + DOX10 group showing a better organization of histological features of renal parenchyma, with mild tubular degenerative changes (red arrow), many apparent intact nephron segments (black arrow), mild tubular dilatation (black star), and minimal interstitial inflammatory cell infiltrates. However, many congested and dilated intratubular BVs persisted (red star). **E** Section taken from a rat in the CP + DOX20 group showing more significant protective efficacy, with abundant records of almost intact renal parenchyma, apparent intact nephron segments *(black arrow)*, mild focal records of degenerative changes and apoptotic body formation *(red arrow),* interstitial tissue, and normal vasculatures. CON, control; CP, cisplatin; DOX, doxepin (scale bar = 50µ = 40 ×)

The renal tissue sections taken from the CON and DOX 20 only treated groups showed well-organized histological features of renal parenchyma with many apparent intact renal corpuscles, different nephron segments with intact lining tubular epithelium, and intact vasculatures. However, the sections taken from rats singly injected with CP showed diffuse necrotic and degenerative changes, including nephron tubular segments with many figures of nuclear pyknosis, occasional records of intraluminal eosinophilic casts and moderate tubular dilatation, and focal interstitial inflammatory cell infiltrates. On the contrary, the DOX co-treatment (5 mg/kg) group showed minimal protective efficacy with persistent records of tubular epithelium degenerative changes, mild tubular dilatation, and many congested and dilated intratubular BVs. However, there were minimal interstitial inflammatory cell infiltrates. In contrast, the DOX co-treatment (10 mg/kg) group demonstrated a better organization of histological features of renal parenchyma, with mild tubular degenerative changes, many apparent intact nephron segments, and mild tubular dilatation accompanied with minimal interstitial inflammatory cell infiltrates. However, many congested and dilated intratubular BVs persisted. Notably, the DOX co-treatment (20 mg/kg) group exhibited more significant protective efficacy, with abundant records of almost intact renal parenchyma with intact nephron segments, mild focal records of degenerative changes and apoptotic body formation, interstitial tissue, and normal vasculatures (Fig. 1). Table 2 shows the histopathological scoring of renal specimens for inflammation, congestion, and degenerative tubular changes of nephron segments for five animals.

**Table 2:** Histopathological scoring of renal specimen for degeneration, congestion, dilatation, inflammation

	Degeneration	Congestion	Cast	Dilatation	Inflammation
CON	0	0	0	0	0
CP	3	3	1	2	3
CP + DOX5	1	2	1	1	1
CP + DOX10	1	2	0	1	1
CP + DOX20	1	0	0	0	1
DOX20 only	0	0	0	0	0

Degeneration, congestion, dilatation, and inflammation were scored as follows: none (0); mild (1); moderate (2); and severe (3). Cast was graded as follows: 0, none; 1, exist. *CON* control, *CP* cisplatin, *DOX* doxepin (*n* = 5)

### *Effect of Doxepin on the expression of organic cationic transporter 2 *via* western blot*

**Fig. 2:**
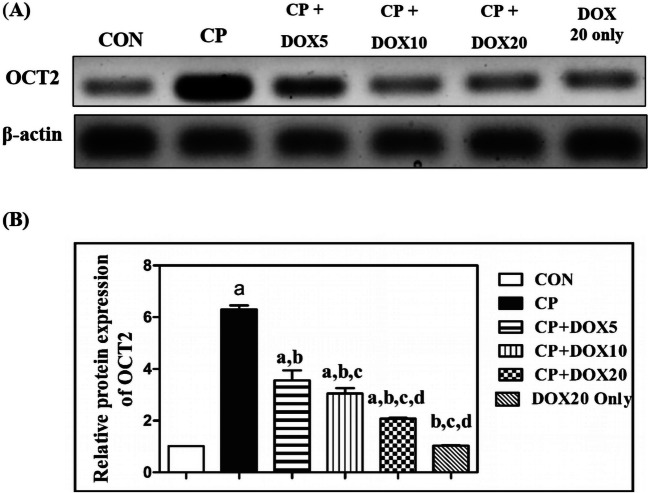
Effect of doxepin on organic cationic transporter 2 expression. **A** Protein expression of OCT2 transporters. **B** Quantitative analysis of OCT2 transporters. Data are presented as mean ± S.D. (*n* = 3). a, b, c, or d: significant difference between the CON, CP, DOX5, or DOX10 groups, respectively, at *p* < 0.05 using ANOVA, followed by the Tukey–Kramer test as a post-hoc test. CON, control; CP, cisplatin; DOX, doxepin; OCT2, organic cationic transporter 2

The nephrotoxic effect of CP and the potential protective effects of DOX as an OCT2 inhibitor were investigated, as shown in Fig. 2. The results of western blot analysis indicated that there was a significant induction of the expression of OCT2 transporter in the CP-administrated group by 6.2-fold as compared to that in the CON group. In contrast, the DOX treatments (5, 10, and 20 mg/kg) markedly decreased this protein expression in a dose-dependent manner by 43%, 51%, and 67%, respectively, as compared to that in the CP group. As compared to the CON values, OCT2 expression in normal rats treated with DOX at 20 mg/kg was not significantly changed. In addition, there was a marked reduction in OCT2 expression in rats co-treated with DOX (10 and 20 mg/kg) by 14% and 42%, respectively, as compared to that in rats co-treated with 5 mg/kg DOX. Further, the co-treatment with 20 mg/kg DOX notably decreased the OCT2 expression by 32% as compared to that in rats co-treated with 10 mg/kg DOX.

### Effect of Doxepin on lipid peroxidation and antioxidant profiles

 Upon CP administration, the renal tissue level of MDA was significantly increased by 4.8-fold as compared to the CON group. In contrast, the administration of 5, 10, and 20 mg/kg of DOX significantly decreased this level by 40%, 49%, and 66%, respectively, as compared to the CP group. Furthermore, compared to rats co-treated with 5 mg/kg DOX, the MDA level in rats co-treated with 10 and 20 mg/kg DOX was significantly reduced by 11% and 43%, respectively, and the co-treatment with 20 mg/kg DOX markedly dropped the MDA level by 33% as compared to that in rats co-treated with 10 mg/kg DOX. However, there was a significant decline in GSH and SOD levels in renal tissues after CP administration by 61% and 68%, respectively, as compared to the CON group. On the other hand, the co-treatment with 5, 10, and 20 mg/kg of DOX significantly increased the GSH levels in a dose-dependent manner by twofold, 2.2-fold, and 2.4-fold, respectively, and the SOD levels by 1.8-, 2-, and 2.6-fold, respectively, as compared to the CP group. There was also a significant rise in GSH and SOD tissue levels by onefold in rats co-treated with 10 mg/kg DOX as compared to that in rats co-treated with 5 mg/kg DOX. There was also a marked elevation in GSH and SOD levels by 1.2-fold and 1.5-fold, respectively, in rats co-treated with 20 mg/kg DOX as compared to those in rats co-treated with 5 mg/kg DOX. Further, 20 mg/kg DOX significantly raised the GSH and SOD levels by 1.1- and 1.3-fold, respectively, as compared to that in rats co-treated with 10 mg/kg DOX. Whereas there was no significant change in tissue levels of MDA, GSH, and SOD between the CON and Dox 20 only treated group (Table 1).

### Effect of Doxepin on inflammatory mediators

CP significantly raised the renal tissue levels of TNF-α, IL-6, IL-2, IL-1β, and MPO by 6.5-fold, 2.8-fold, threefold, 3.8-fold, and 2.2-fold, respectively, as compared to those in the CON group. On the other hand, the administration of DOX (5, 10, and 20 mg/kg) dose-dependently induced a significant decline in the levels of TNF-α by 35%, 46%, and 72%, respectively, IL-6 by 38%, 45%, and 53%, respectively, IL-2 by 37%, 46%, and 61%, respectively, IL-1β by 37%, 44%, and 59%, respectively, and MPO by 30%, 39%, and 49%, respectively, as compared to those in the CP group, while there was not a significant change in tissue levels of TNF-α, IL-6, IL-2, IL-1β, and MPO in normal rats treated with DOX (20 mg/kg) and CON rats. Further, there was a marked reduction in TNF-α, IL-6, IL-2, IL-1β, and MPO levels by 17%, 11%, 15%, 12%, and 13%, respectively, in rats co-treated with 10 mg/kg Dox as compared to those in rats co-treated with 5 mg/kg DOX. Moreover, the co-treatment with 20 mg/kg DOX significantly declined the TNF-α, IL-6, IL-2, IL-1β, and MPO levels by 57%, 25%, 38%, 35%, and 28%, respectively, as compared to those in rats co-treated with 5 mg/kg DOX. Additionally, there was a significant decline in TNF-α, IL-6, IL-2, IL-1β, and MPO levels in rats co-treated with 20 mg/kg DOX by about 49%, 15%, 27%, 26%, and 16%, respectively, as compared to those in rats co-treated with (10 mg/kg) DOX (Table 3).

**Table 3:** Ameliorating effect of DOX on inflammatory indicators

Treated groups	TNF-α (pg/mg protein)	IL-1β (pg/mg protein)	IL-6 (pg/mg protein)	IL-2 (pg/mg protein)	MPO (pg/mg protein)
CON	11.65 ± 2.012	34.30 ± 3.16	65.30 ± 6.003	83.85 ± 5.73	225.8 ± 15.28
CP	76.30 ± 3.069^a^	131.1 ± 4.11^a^	185.1 ± 4.702^a^	263.7 ± 8.33^a^	504.5 ± 15.09^a^
CP + DOX5	49.28 ± 6.49^a,b^	82.80 ± 9.62^a,b^	115.2 ± 14.03^a,b^	167.2 ± 13.50^a,b^	353.3 ± 47.13^a,b^
CP + DOX10	41.00 ± 3.33^a,b,c^	73.10 ± 3.41^a,b,c^	102.1 ± 3.43^a,b,c^	142.3 ± 11.40^a,b,c^	306.1 ± 10.43^a,b,c^
CP + DOX20	21.15 ± 2.67^a,b,c,d^	54.05 ± 3.21^a,b,c,d^	86.50 ± 5.03^a,b,c,d^	104.0 ± 7.34^a,b,c,d^	256.3 ± 12.06^b,c,d^
DOX 20 only	15.32 ± 2.24^b,c,d^	37.05 ± 4.07^b,c,d^	56.40 ± 4.33^b,c,d^	94.85 ± 7.79^b,c,d^	213.4 ± 10.65^b,c,d^

Rats were received DOX (5, 10, 20 mg/kg, ip) for 10 days. On the 7th day, rats were administrated a single injection dose of CP (10 mg/kg, ip). Data are represented as mean ± SD (*n* = 6). a, b, c or d: significant difference from CON, CP, DOX5, or DOX 10 groups, respectively at *p* < 0.05 using ANOVA followed by Tukey–Kramer as a post-hoc test. *CON* control, *CP* cisplatin, *DOX* doxepin, *TNF-α* tumor necrosis factor- α, *IL-1β* interlukin-1β, *IL-6* interlukin-6, *IL-2* interlukin-2, *MPO* myeloperoxidase

### Effect of Doxepin on the phosphorylation of the PI3K/Akt pathway

**Fig. 3:**
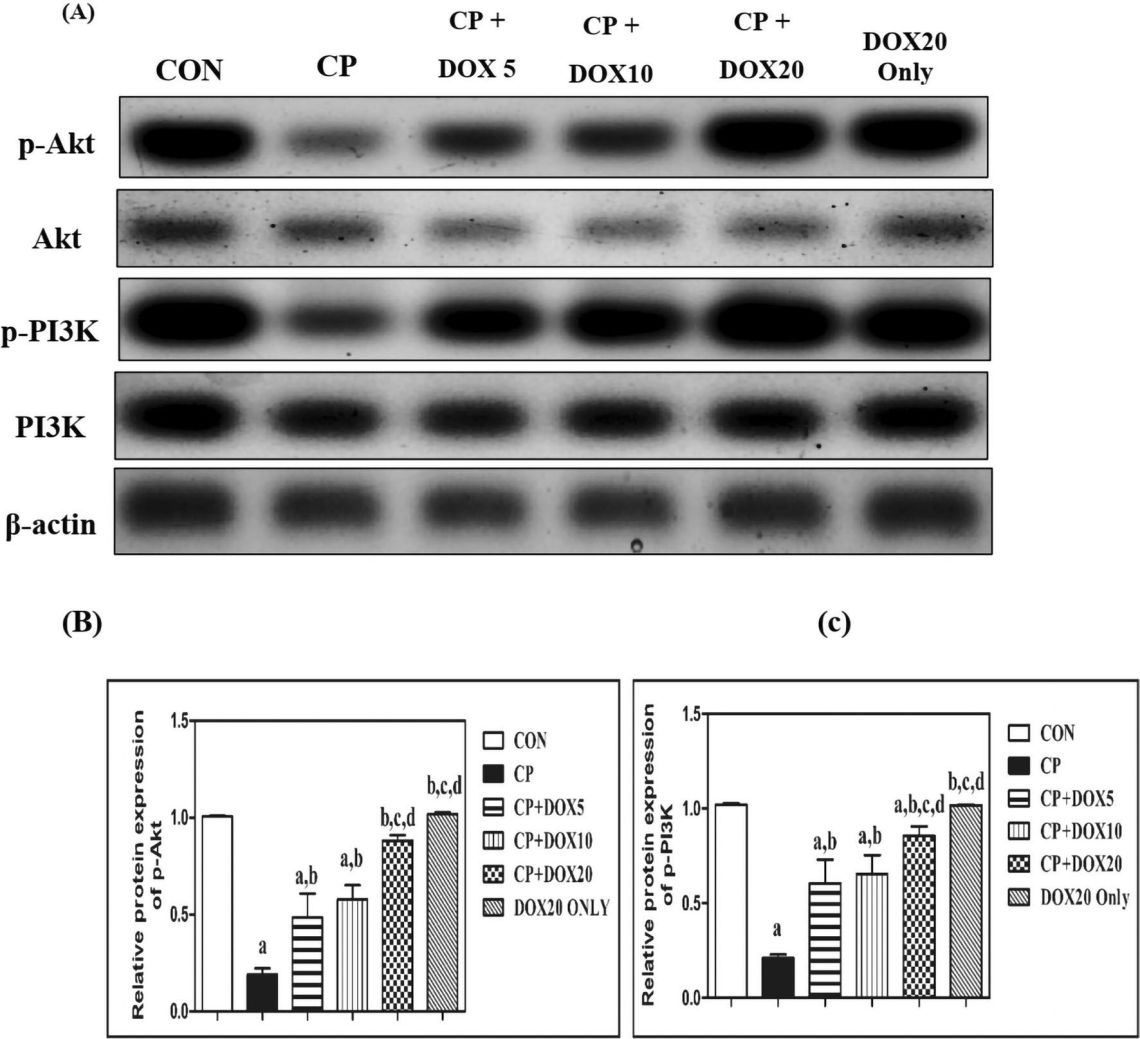
*Effect of doxepin on the phosphorylation of the PI3K/Akt signaling cascade against the nephrotoxic impact induced by cisplatin*. **A** Protein expression of PI3K/Akt. **B** Quantitative analysis of PI3K/Akt. Data are presented as mean ± S.D. (*n* = 3). a, b, c, or d: Significant difference between the CON, CP, DOX5, or DOX10 groups, respectively, at *p* < 0.05 using ANOVA, followed by the Tukey–Kramer test as a post-hoc test. CON, control; CP, cisplatin; DOX, doxepin; Akt, protein kinase B; p-Akt, phosphor-Akt; PI3K, phosphatidylinositol 3-kinase

PI3K/Akt signaling pathway employs a nephroprotective effect versus nephrotoxic damaging effect of CP. Figure 3 shows that CP significantly reduced the expression of PI3K and Akt by 79% and 81%, respectively, as compared to the CON group, expressed by western blot results revealing the renal injury. On the other hand, the co-administration of DOX (5, 10, 20 mg/kg) dose-dependency restored the CP-induced decline in the expression of PI3K by 2.9-, 3-, and fourfold, respectively, and that of Akt by 2.5-, 3-, and 4.6-fold, respectively, as compared to the CP group, while the expression of PI3K and Akt was not significantly changed in normal rats treated with DOX (20 mg/kg) as compared with the CON group. Indeed, it was observed that rats co-treated with (20 mg/kg) DOX expressed a marked enhancement in the expression of PI3K and Akt by about 1.4- and 1.8-fold, respectively, as compared to those in rats co-treated with (5 mg/kg) DOX. Moreover, (20 mg/kg) DOX significantly raised the PI3K and Akt expression by 1.3- and 1.5-fold, respectively, as compared to those in rats co-treated with (10 mg/kg) DOX.

### Doxepin counteracts the mitochondrial apoptotic effect of cisplatin via the modulation of Bcl-2 family members

**Fig. 4:**
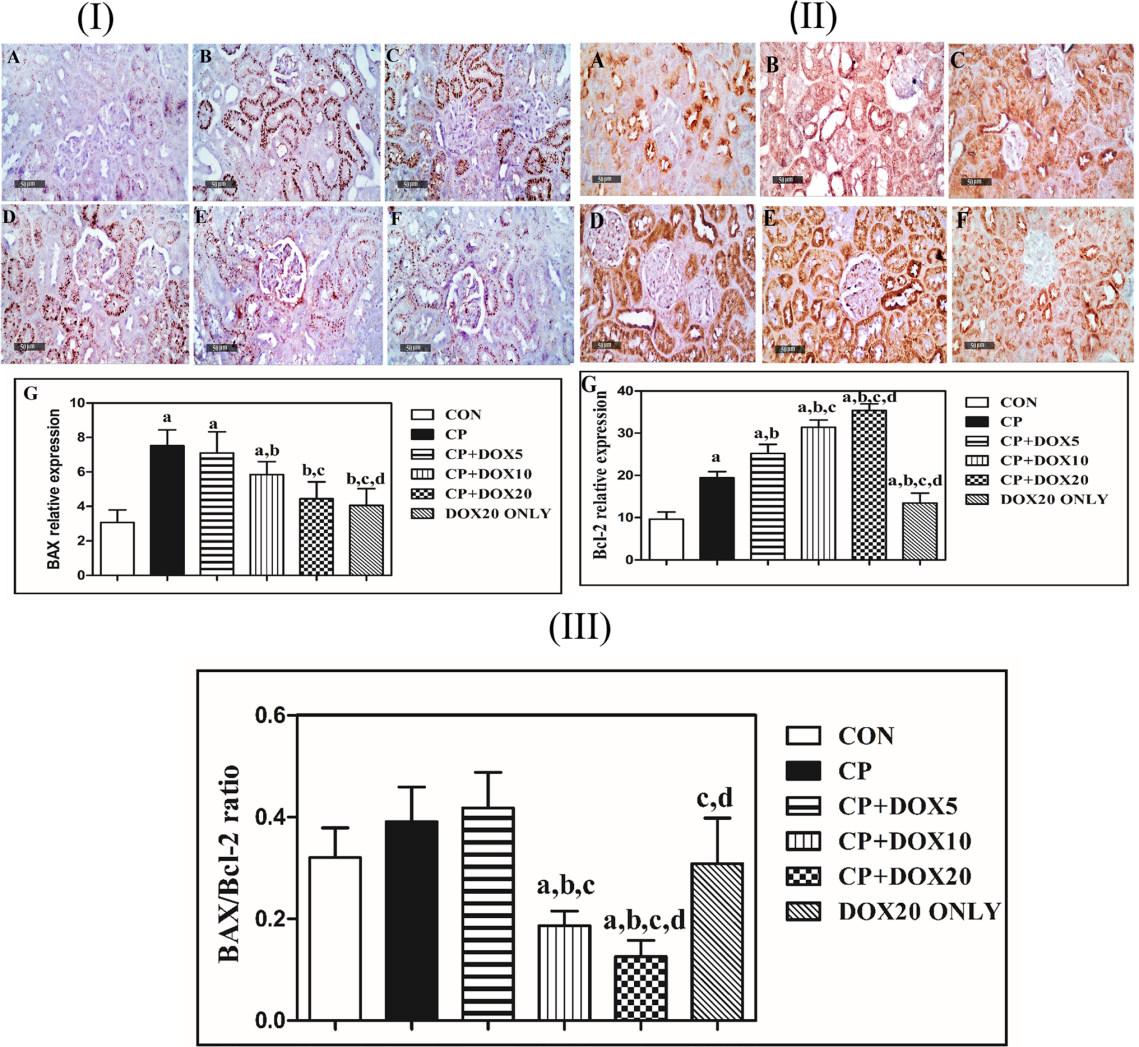
Immunohistochemical staining of the effects of doxepin on cisplatin-induced apoptotic effects via the alteration of the expression of Bcl-2 family members. BAX expression was enhanced by the administration of CP (intense brown color), but it was reduced by the concomitant treatment with DOX, which is shown as a mild to moderate reduction in brown color (I). Conflicting findings about the Bcl-2 expression were revealed (II). The BAX/Bcl-2 ratio was increased in the CP group, and this rise was amended in DOX-co-treated groups (III). **A** Section taken from the CON group. **B** Section taken from the CP group. **C** Section taken from rats administrated with CP and co-treated with 5 mg/kg DOX. **D** Section taken from rats administrated with CP and co-treated with 10 mg/kg DOX. **E** Section taken from rats administrated with CP and co-treated with 20 mg/kg DOX. **F** *Area* percentage of immunostaining in the renal tissues of the different studied groups. Data are presented as mean ± S.D. (*n* = 5). a, b, c, or d: Significant difference between the CON, CP, DOX5, or DOX10 groups, respectively, at *p* < 0.05 using ANOVA, followed by the Tukey–Kramer test as a post-hoc test. *CON*, control; *CP*, cisplatin; *DOX*, doxepin; *BAX*, bcl-2-like protein 4*; Bcl2,* B-cell lymphoma

As shown in Fig. 4, the immunohistochemical staining of renal tissues taken from the CP group revealed a significantly increased BAX expression by 2.5-fold and increased Bcl-2 expression by twofold, leading to elevated BAX/Bcl-2 ratio by 1.2-fold as compared to that in the CON group. In contrast, the co-administration of DOX (5, 10, and 20 mg/kg) in a dose-dependent manner significantly decreased the BAX expression by 5.5%, 22%, and 41%, respectively, and markedly boosted the Bcl-2 expression by 1.3-, 1.6-, and 1.8-fold, respectively, as compared that in the CP group. This resulted in a notable increase in BAX/Bcl-2 ratio by 1.1-fold in rats co-treated with 5 mg/kg DOX and in marked reduction in BAX/Bcl-2 ratio by 52% and 68% in rats co-treated with (10 and 20 mg/kg) DOX, respectively. Besides, as compared with the CON group, there was not a significant change in BAX expression in normal rats treated with DOX (20 mg/kg). Despite, DOX-co-treated normal rats (20 mg/kg) significantly displayed a marked rise in Bcl-2 expression by 1.4-fold, as compared to CON rats. However, there was not a significant change induced in the BAX/Bcl-2 ratio. Further, the co-treatment with (10 mg/kg) DOX significantly augmented the Bcl-2 expression by about 1.3-fold, but it did not significantly change the BAX expression, leading to a decline in BAX/Bcl-2 ratio by 55% as compared to that in rats co-treated with 5 mg/kg DOX. Moreover, the rats co-treated with (20 mg/kg) DOX displayed a significant decrease in BAX expression by about 38% and increase in Bcl-2 expression by (1.4-fold), resulting in a significant drop in BAX/Bcl-2 ratio by 70% as compared to that in rats co-treated with (5 mg/kg) DOX. Moreover, there was a significant decrease in BAX expression 24% and a marked increase in Bcl-2 expression by 1.2-fold in rats co-treated with 20 mg/kg DOX as compared to those in rats co-treated with 10 mg/kg DOX, leading to a notable reduction in BAX/Bcl-2 ratio by about 33%.

### Modulating effect of Doxepin on caspase -3 activation

**Fig. 5:**
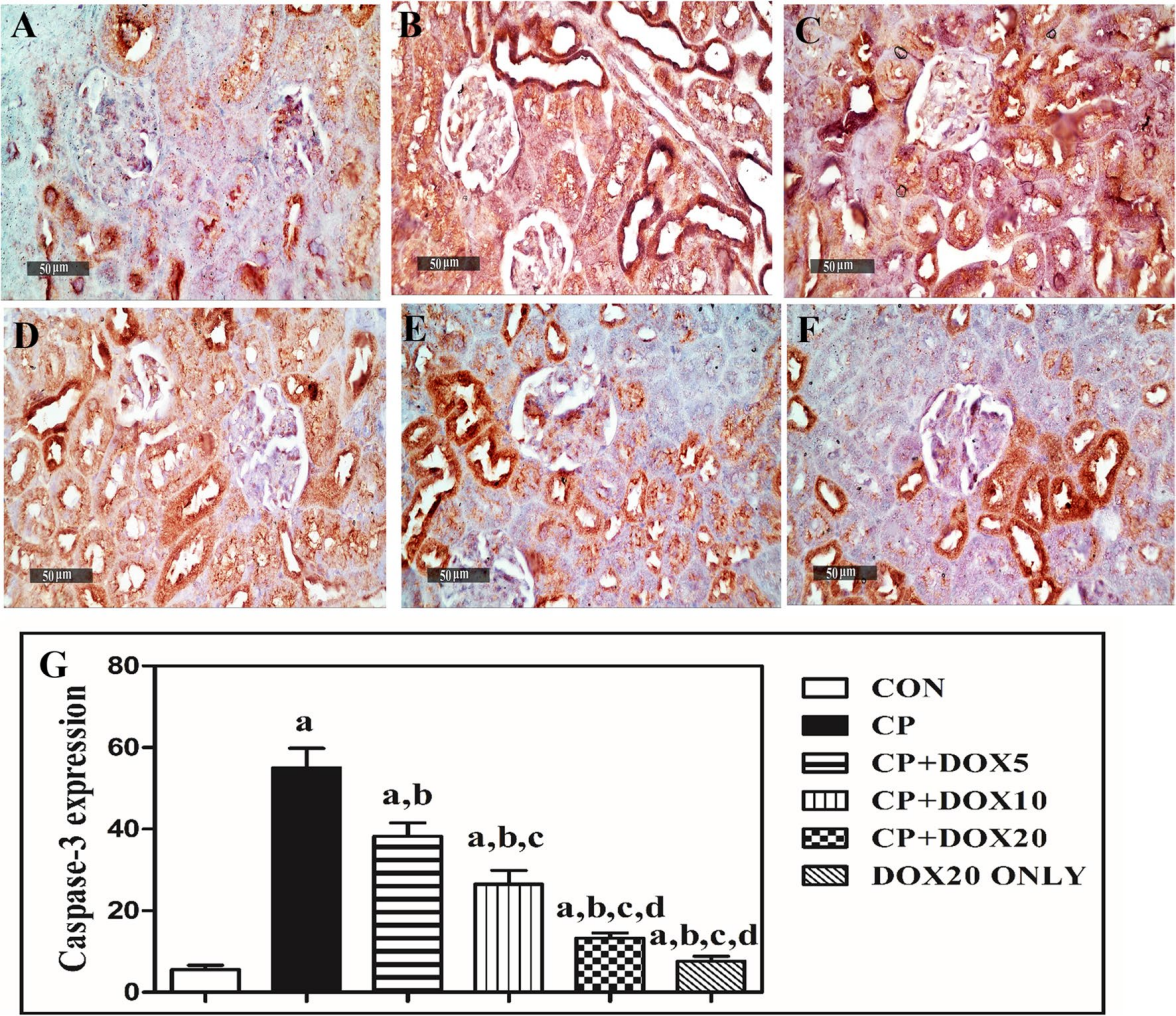
Immunohistochemical staining of the effects of doxepin on cisplatin-mediated caspase-3 activation. **A** Section taken from the CON group showing minimal immunostaining. **B** Section taken from the CP group showing extensive immunostaining (brown color). **C** Section taken from rats administrated with CP and co-treated with 5 mg/kg DOX showing moderate immunostaining. **D** Section taken from rats administrated with CP and co-treated with 10 mg/kg DOX showing minimal immunostaining. **E** Section taken from rats administrated with CP and co-treated with 20 mg/kg DOX showing limited immunostaining. **F** Area percentage of immunostaining in the renal tissues of the different studied groups. Data are presented as mean ± S.D. (*n* = 5). a, b, c, or d: Significant difference between the CON, CP, DOX5, or DOX10 groups, respectively, at *p* < 0.05 using ANOVA, followed by the Tukey–Kramer test as a post-hoc test. CON, control; CP, cisplatin; DOX, doxepin

Compared to the CON group, CP markedly enhanced the caspase-3 expression by tenfold, but the co-treatment with 5, 10, and 20 mg/kg DOX decreased this expression by 31%, 52%, and 76%, respectively, as compared to the CP group. Besides, caspase-3 expression was not significantly changed in normal rats treated with DOX (20 mg/kg) and CON group. Indeed, the co-treatment with DOX (10 and 20 mg/kg) markedly decreased the caspase-3 expression by 31% and 65%, respectively, as compared to that in rats co-treated with (5 mg/kg) DOX. There was also a significant decrease in caspase-3 expression in rats co-treated with (20 mg/kg) DOX by about 50%, as compared to that in rats co-treated with (5 mg/kg) DOX (Fig. 5).

## Discussion

This study aimed to look into the potential protective effect of DOX against the nephrotoxic effect of CP, including the processes underlying it. It was reported that DOX exerts a modulating effect on the phosphorylation of PI3K/Akt signaling cascade. The PI3K/Akt signaling cascade has been reported to be involved in improving the inflammatory (Wu et al. 2017), oxidative (Liu et al. 2014), and apoptotic states (Zhang et al. 2018). In particular, the inhibitory impact of DOX on OCT2 transporters has been corroborated by prior research and has attracted a lot of attention (Noorlander et al. 2021). To investigate the most beneficial nephroprotective dose of DOX, three doses of DOX (5, 10, and 20 mg/kg) were tested.

It was demonstrated that CP becomes concentrated and even raises its toxic level in renal tubular cells to around fivefold greater than its blood level (Ozkok and Edelstein 2014). This occurrence was linked to a change in kidney function, which was reflected in Scr, BUN, and albumin level alterations (Palipoch and Punsawad 2013). In this study, there was a significant increase in Scr and BUN levels and a significant decrease in albumin levels subsequently after injecting a single dose of CP in accordance with a previous study (Ismail et al. 2020). On the contrary, an increase in DOX dose resulted in a significant decrease in Scr and BUN levels with modulation of albumin levels, expressing the improvement in all kidney function indicators. Besides, the results of the histopathological examination of the renal tissues of rats treated with CP showed diffuse necrotic and degenerative changes, including nephron tubular segments with many figures of nuclear pyknosis and focal interstitial inflammatory cell infiltrates in correlation with another study (Domitrović et al. 2013). Conversely, the dose-dependent effect of DOX presented by the significant protective efficacy with abundant almost intact renal parenchyma with intact nephronal segments, mild focal records of degenerative changes and apoptotic body formation, interstitial tissue, and normal vasculatures suggests the ameliorating effects of DOX on renal tissues.

Indeed, CP acts as a substrate for OCT2, exerting a selective and higher sensitivity for nephrotoxicity compared to the CP analog cardiolipin, which showed less specificity for OCT2 and less nephrotoxic effects on cells (Yonezawa and Inui 2011). Other related studies claimed that there was a boost in OCT2 expression associated with CP-induced nephrotoxicity (Filipski et al. 2008). It was revealed that knocking out this vital transporter has a protective effect on the nephrotoxicity of CP without affecting its critical antineoplastic impact, as supported by another related study that demonstrated the absence of OCT2 transporters in various human neoplasms, implying that OCT2 plays a critical role in evoking nephrotoxicity (Sprowl et al. 2013). In line with other reports, CP displayed increased the expression of OCT2, as shown in western blot findings (Franke et al. 2010). On the contrary, the co-treatment with DOX evidently decreased the OCT2 expression in a dose-dependent manner, showing the ameliorating impact of DOX as an OCT2 inhibitor, which was also shown in another study that presented DOX as an important OCT2 inhibitor (Noorlander et al. 2021).

Mitochondria are known to be the most susceptible area to CP nephrotoxic effect (Yu et al. 2018). This was justified previously by the reduction in its ability to repair its DNA effectively. Moreover, the rich density of mitochondria in RPTECS justified the certain sensitivity of S3 segments to the toxic effects of CP (Hall and Schuh 2016). The great propensity of CP to preferentially target the mitochondria ends with the accumulation of CP that contributes to the induction of ROS and impairs the defensive antioxidant activity (Miller et al. 2010). This acts as a vital event in the initiation of lipoperoxidation of mitochondrial lipids, manifested by the increase in MDA levels and decrease in GSH and SOD levels. Similar to earlier research, the current study revealed that there was a significant increase in MDA levels and significant drop in GSH and SOD levels, demonstrating the toxic effects of CP in rats (Okafor 2021). Conversely, the co-treatment with DOX markedly decreased the MDA levels, restored GSH, and maintained all antioxidant enzyme activities of SOD in a dose-dependent manner, indicating the antioxidant effects of DOX against the toxic effects of CP in renal tissues. These antioxidant effects of DOX were supported by previous studies (Ji et al. 2004). In a recent study, it was demonstrated that in cases of nonalcoholic fatty liver disease and diabetes, DOX may worsen the renal injury. This action of DOX is probably attributed to the existence of insulin resistance and obesity that both play a role in alternating inflammatory and antioxidant enzyme levels (Chang et al. 2021). Therefore, there is a need for further research to investigate this conflict.

Further, the CP-mediated generation of ROS contributes mainly to the release of proinflammatory TNF-α, which is considered a critical modulator of additional inflammatory mediators, such as IL-1β, IL-6, IL-2, and MPO, which have also been implicated in CP-induced nephrotoxicity, exacerbating the renal tissue injury and possibly leading to apoptosis (Yan et al. 2017; Karadeniz et al. 2011). Consistent with a previous study (Domitrović et al. 2013), the current study showed that rats injected with CP exhibited a marked rise in all these abovementioned inflammatory markers. In contrast, the co-treatment with DOX markedly attenuated this inflammatory status in a dose-dependent manner, demonstrating a significant decrease in TNF-α, IL-1β, IL-6, IL-2, and MPO tissue levels, indicating the anti-inflammatory effects of DOX ameliorating the nephrotoxicity mediated by CP. These results supported the findings of earlier studies that illustrated the crucial effects of DOX on the inflammatory cytokines in inflammatory paw edema mediated via carrageenan (Zabihi et al. 2017). These findings suggested the probable nephroprotective effects of DOX as an anti-inflammatory drug.

The previous study has clarified that CP-induced irreversible renal injuries are attributed to excessive apoptosis and alterations in the PI3K/Akt pathway (Zhang et al. 2018). The PI3K/Akt signaling cascade is involved in various key cellular processes involving proliferation and differentiation (Miao and Degterev 2011). The PI3K/Akt pathway also plays an important role against inflammation based on its ability to inhibit the activation of the NF-κB pathway and its related corresponding inflammatory contributors (Gong and Wang 2018). In line with these previous findings, the results of the present study showed a notable decrease in PI3K/Akt phosphorylation after the exposure to CP, as supported by the detection of PI3K/Akt expression in western blot (Torigoe et al. 2020). In contrast, there was a marked increase in PI3K/Akt phosphorylation following the DOX treatment. Previous studies suggested the modulatory effects of DOX on the PI3K/Akt cascade versus neuroinflammatory diseases mediated via lipopolysaccharides in C6 glioma cells (Tao et al. 2020). The improvement in the phosphorylation was in line with the increase in the dose of DOX. Collectively, the findings of the current study suggest the antioxidant, anti-inflammatory, and anti-apoptotic effects of DOX via the modulation of the PI3K/Akt pathway.

Bcl-2 family proteins are involved in intrinsic mitochondrial pathways by which CP evokes nephrotoxicity and apoptotic renal injury (dos Santos et al. 2012). Mounting evidence underlines the influential role of CP in triggering the BAX activity that results in the liberation of cytochrome C and the upregulation of apoptotic caspase-3 activity (Liu et al. 2014). The role of Bcl-2 in the suppression of BAX translocation to the mitochondria has also been illustrated, suggesting its key anti-apoptotic effect (Fan and Fan 2017). In this study, there was a rise in BAX and caspase-3 expression after the administration of CP, exacerbating cell death, in line with the previous study (Domitrović et al. 2013). There was also a notable increase in Bcl-2 expression similar to previous studies, which related this rise to the absence of alterations in Bcl-2 expression level in response to CP, while there was a marked amplification in BAX expression, as supported by earlier studies (Gao et al. 2013). In contrast, in accordance with the previous study (Meng et al. 2019; Reisi et al. 2017), DOX markedly suppressed the enhancement in BAX and caspase-3 expression, while increased that of the Bcl-2 in a dose-dependent manner, suggesting the potential protective anti-apoptotic effects of DOX. These results supported earlier studies that illustrated the vital anti-apoptotic effects of DOX (Drzyzga et al. 2009).

## Conclusion

In summary, this current study showed the antioxidant, anti-inflammatory, and anti-apoptotic impact of DOX on CP-induced nephrotoxicity by inhibiting OCT2, reducing the levels of MDA, which is an oxidative status indicator, and increasing the antioxidant activity of GSH and SOD. In addition, DOX significantly decreased the surge of other inflammatory cytokines. DOX also markedly decreased the relative BAX/Bcl-2 ratio and raised the Bcl-2 expression. DOX also showed a protective effect on renal tissues based on histopathological results, which showed its impact as a modulator of the PI3k/Akt signaling cascade. The results of this study showed that DOX is a promising agent against the nephrotoxic impact of CP.

## Data Availability

No datasets were generated or analysed during the current study.

## Declarations

### Research involving animals

This study was carried out according to the “Guidelines of the Animal Care and Use of Laboratory Animals” and in parallel with the guidelines adopted by the Research Committee at Suez Canal University (approval no. 201911PHDA2).
